# A Nutraceutical Approach Using Herbs, Vitamins, Trace Elements, and Amino Acids for the Treatment of Insomnia Disorder and Anxiety: An Eight-Week Observational Study

**DOI:** 10.7759/cureus.79303

**Published:** 2025-02-19

**Authors:** Gianluca Bruti, Paola Di Giacomo, Alice Pratesi, Carlo Di Paolo

**Affiliations:** 1 Neurology and Psychology, Eurekacademy, Center for International Studies of Cognitive Neurosciences and Integrated Medicine, Rome, ITA; 2 Oral and Maxillofacial Sciences, Sapienza University of Rome, Rome, ITA; 3 Psychology, Eurekacademy, Center for International Studies of Cognitive Neurosciences and Integrated Medicine, Rome, ITA

**Keywords:** anxiety, dietary supplements, hypervigilance, insomnia disorder, non-pharmacological approach, sleep-wake cycle

## Abstract

Objectives

This prospective, observational, single-arm, open-label study aimed to evaluate the efficacy and safety of a phytotherapeutic and nutraceutical compound in the treatment of patients with insomnia disorder (ID) associated with anxiety.

Methods

The study was conducted on a clinical sample of 28 patients (6 men, 21.4%, and 22 women, 78.6%) suffering from ID, associated with a state of anxiety.

The sample study was clinically evaluated at baseline (T0) and after four (T1) and eight weeks (T2) of phytotherapeutic and nutraceutical treatment using the following self-administered questionnaires: Pittsburgh Sleep Quality Index, State and Trait Anxiety Inventory, Insomnia Severity Index, Depression and Anxiety Scale Short Form - 21, Beck Depression Inventory, Fatigue Severity Scale, Rapid Stress Assessment, Patient Global Impression of Improvement - Severity and short form-36.

Results

The effect of treatment over time was statistically significant for all measures considered including the short form-36 subscales 3 and 5, with the exception of the other short form-36 subscales and fatigue severity scale.

Linear regression reported a significant association only between T1 and T2 scores (p<0.05). No correlations were found with age except for the test short form-36 subscale 5 (Pearson r=0.421; p =0.036 and tau Kendal =0.311; p=0.033) and no correlation for the level of education. No safety concerns were reported in the sample study.

Conclusion

The results of this study support the clinical efficacy and safety of the phytotherapeutic and nutraceutical study compound for the treatment of ID in patients with anxiety symptoms.

## Introduction

Over the past two decades, research has changed the clinical and scientific meaning of sleep disorders, transforming them from a symptom of other pathologies to a disease in its own right.

In this regard, the definition of insomnia disorder (ID) provided in the Diagnostic and Statistical Manual of Mental Disorders (DSM-V) [1] clearly describes a specific and independent illness that can have a negative impact on the functioning of the affected patient.

By overcoming the old paradigm that differentiated primary from secondary insomnia, this new definition of insomnia indirectly highlights the importance of clinical phenotypes and pathophysiological mechanisms in choosing the most appropriate approach for the treatment of ID.

In this perspective, emotional and psychological factors play an important role in predisposing, precipitating, and perpetuating ID [2]. Indeed, comorbidity between sleep disorders and mental and physical disturbances has been widely demonstrated, suggesting a common pathophysiological mechanism underlying these associated pathologies [3]. In fact, the mechanisms on the basis of the bidirectional link between sleep disturbances and anxiety disorders could represent a key factor in the decision-making process for treatment of sleep disorders [4]. As a matter of fact, sleep disorders and anxiety disorders, in addition to constituting a complex syndrome, represent a clinical condition in which various interconnected pathophysiological mechanisms contribute to determining the clinical phenotype. One of the most plausible pathophysiological models that can explain this comorbidity and the related nocturnal and daytime symptoms is the hyperarousal model of ID [5]. According to this model, ID may be caused by an increased level of arousal secondary to a dysfunction of the bodily stress system with hyperactivation of both the sympathetic nervous system and hypothalamus-pituitary-adrenal axis [6]. In other words, and more specifically, ID should be considered a night and day disorder characterized by a state of hypervigilance associated with a high "inflammation tone" in a subject with anxious-ruminative personality traits with a positive history of multiple traumatic stressful life events. From this perspective, the therapeutic strategy in ID should be to restore the balance in the psychological, neurophysiological, immunological, and endocrine components of the hyperarousal system throughout the 24-h sleep/wake period.

Indeed, despite significant progress in understanding the pathophysiology of sleep disorders, cognitive behavioral therapy for insomnia (CBT-I) remains the first line of treatment for ID although it is currently poorly available in real-world clinical practice also in its digital form [7].

On the other hand, for the above-reported data, the ideal drug in patients with ID, in addition to having an anxiolytic and hypno-inducing action, should have a modulatory action on chronic stress, mood, and inflammatory state, with a therapeutic action also on the cognitive and physical dimensions of the clinical syndrome. What is certain is that the pharmacological treatment of sleep disorders remains a clinical challenge due to numerous warnings that available drugs have. In this view, the recent European Insomnia Guideline (EIG), underlying the cognitive and psychological side effects they may have, does not support the long-term use (more than four weeks) of benzodiazepines (BZs) and benzodiazepine receptor agonists (BZRAs) for the treatment of ID [7]. In fact, in many cases, the widespread use in clinical practice of BZs and BZRAs could represent an even iatrogenic barrier to the healing process of ID, leading to dependence, withdrawal syndromes, sedation, and memory impairment. On the other hand, the so-called z-drugs must be considered symptomatic agents not capable of re-establishing the balance of the organism’s vigilance and stress systems.

Among other pharmacological compounds, sedating antidepressants like trazodone and doxepin have demonstrated a modest level of efficacy, but their off-label use is recommended, as also suggested for BZs and BZRAs, for short-term treatment (up to four weeks) and in patients with comorbid depression [7]. Furthermore, antipsychotics and antihistamines commonly used in clinical practice in patients with psychiatric comorbidity have no indication for ID due to the lack of dedicated randomized clinical trials and the potential safety concerns [7].

A separate topic in the field of pharmacological treatment for ID is represented by dual orexin receptor antagonists (DORAs), a class of agents capable on the one hand of blocking the orexin receptor by exerting an inhibitory effect on a sleep center called the ventrolateral preoptic area, and on the other hand of stabilizing the state of wakefulness and vigilance based on the life context [7]. It is important to underline that the mechanism of action of these compounds allows them to be taken beyond the classic four-week period, extending the duration of treatment up to three months and on an individual basis even longer [7]. Indeed, the clinical rationale for the use of DORAs is based on their ability to regulate hyperarousal and hypervigilance states which are hypothesized to be responsible for the dysfunction of the light/dark cycle in ID. Nevertheless, the EIG stated that further pharmacovigilance and phase four studies are needed to establish the appropriate cost-benefit ratio of this new class of drugs for ID, particularly for long-term treatment [7].

The lack of quality and the low level of evidence shown by studies conducted with herbs and phytotherapeutic extracts in patients with sleep disorders do not allow physicians to recommend this treatment approach to date [7]. For these reasons, more in-depth clinical studies are needed in this field, using a combination of more natural compounds, specifically designed based on the dimensional and phenotypic pattern of the disease.

According to the hyperarousal model of ID, a dietary supplement dedicated to a population of patients affected by this disorder associated with specific and non-specific states of anxiety could be made up of four distinct therapeutic principles (plant extracts, amino acids, vitamins, minerals) with a reinforcing action on the physiological mechanisms responsible for regulating the sleep-wake rhythm and the homeostasis of the neurotransmitter and immune systems involved in the regulation of the state of alertness, mood and the hypothalamic-pituitary-adrenal axis. The therapeutic rationale is based on the principle of the synergy of action exerted by the constituents. These include plant extracts, which directly act on GABA-A receptors implicated in the induction of sleep and address mechanisms of chronic inflammation; precursor amino acids, which contribute to the synthesis of key substances such as γ-aminobutyric acid (GABA), melatonin, dopamine, and serotonin; vitamins, which act as enzymatic catalysts in the synthesis of molecules involved in the regulation of circadian rhythms and mood states; and minerals, which serve as essential cofactors for the correct functioning of neurotransmitters, neuro-hormones, and related chemical reactions. These mechanisms align with the multidimensional approach of the study, targeting both the physiological and psychological aspects of insomnia and anxiety.

The aim of the present study was to evaluate the efficacy and safety of a natural multicomponent compound for the treatment of a clinical sample affected by ID associated with anxiety. In particular, using a multidimensional model, we aimed to demonstrate the efficacy of this compound in improving not only the clinical variables related to sleep disturbance itself but also several clinical aspects of ID such as anxiety, depressive symptoms, perception of stress, fatigue, and quality of life. Furthermore, to evaluate the multidimensional effect of this natural compound over time, the study was conducted over a period of eight weeks.

## Materials and methods

This was a prospective, observational, eight-week, single-center, single-arm, open-label study conducted on patients with ID associated with anxiety symptoms, recruited consecutively from September 2021 to September 2022. The inclusion and exclusion criteria are detailed in Table 1, listing conditions required for patient eligibility. Eligibility for the study was verified at two time points: during the initial screening phase, where patients were assessed using the Insomnia Severity Index (ISI) [8] and the State and Trait Anxiety Inventory (STAI-Y1/Y2) [9], and during the first recruitment visit when all inclusion and exclusion criteria were confirmed. All participants provided written informed consent prior to enrollment in the study. The validated psychometric tools used ensured that anxiety symptoms were measured with precision, focusing on symptom severity rather than requiring a clinical diagnosis of anxiety. This approach allowed us to identify and include patients experiencing significant anxiety symptoms associated with ID, while excluding those with comorbidities such as substance use disorders or ongoing hypno-inducing drug therapies, to maintain a homogeneous study population.

**Table 1:** The inclusion and exclusion criteria of the study.

Inclusion Criteria	Exclusion Criteria
Informed consent	Hypno-inducing drug therapy
Men and women	Positive history for substance abuse disorders
Insomnia disorder (DSM-V) and insomnia severity index > 8 [8]	Pregnancy or breastfeeding
Anxiety state (state and trait anxiety inventory) > 40 [9]	Workers with night shifts
Sleep time: between 08.30 and 12.00 PM	Body mass index > 16.5-30 kg/m^2^
Time needed to fall asleep: > 30 minutes	Positive history for epilepsy and head trauma
Number of hours of sleep per night: < 6,5	Unstable biopsychosocial status
Stable neuropsychiatric treatment with psychotropic drugs for at least three months	Unstable neuropsychiatric clinical syndrome

According to these criteria, patients had to present with ID associated with anxiety and a stable clinical state to participate in the eight-week study (Table 1).

Table 2 shows the validated self-administered questionnaires that were used during the study to evaluate the presence and severity of the sleep disorder, together with other clinical symptoms often associated with it such as anxiety disorder, depressive disorder, symptoms related to stress and fatigue. More specifically, among other psychometric measures, we administered the Pittsburgh Sleep Quality Index (PSQI) [10] for comprehensive sleep assessment, the Beck Depression Inventory (BDI) [11] for mood assessment, the Depression and Anxiety Scale-Short Form - 21 (DASS-21) [12] for alternative measures of mood and anxiety, the Rapid Stress Assessment (RSA) [13] for subjective perception of stress, the Fatigue Severity Scale (FSS) [14], the 36-Item Short Form for the evaluation of quality of life [15], and the Patient's Global Impression of Insomnia Severity (PGI-S) [16] (Table 2).

**Table 2:** Self-administered questionnaires of the study with the relevant clinical meaning.

Self-administered Questionnaire	Clinical Meaning
Pittsburgh sleep quality index (PSQI) [10]	Sleep quality
Insomnia severity index (ISI) [8]	Sleep severity
State and trait anxiety inventory (STAI-Y1/Y2) [9]	The symptomatology of anxiety as state and trait
Beck depression inventory (BDI) [11]	Depressive symptoms
Depression and anxiety scale short-form – 21 (DASS-21) [12]	Anxiety, depressive, and stress-related symptoms
Rapid stress assessment (RSA) [13]	Stress-related symptoms
Fatigue severity scale (FSS) [14]	Fatigue-related symptoms
Short form – 36 [15]	Measurements of functioning and disability
Patient global impression – s (PGI-S) [16]	Subjective severity measure of insomnia

Each questionnaire was administered at baseline (T0), four weeks of treatment (T1), and eight weeks of treatment (T2) for comparative analysis of the efficacy of the compound during the treatment period.

Participants received a single daily sachet containing a pre-mixed combination of all active ingredients (Table 3). The contents were dissolved in water and consumed approximately one hour before sleep for eight consecutive weeks. This combined formulation was developed specifically for this study and aimed to leverage the synergistic effects of its components. 

**Table 3:** Active ingredients, dosages, indications, and pharmacodynamics of compounds used in the study. GABA: γ-aminobutyric acid; NMDA: N-methyl-D-aspartate

Therapeutic Principles	Dosages (mg/die)	Active Ingredients	Indication	Pharmacodynamics
Plant				
Valeriana officinalis L.	200 mg	Valerenic acid (0.8%)	Anxiety/Insomnia	GABA-A, melatonin, and adenosine receptor modulation; partial agonist of the 5-HT5A receptor
Ashwagandha	150 mg	Glycovitanolides (35%) for removal of alkaloids	Anxiety/Insomnia	HPA axis regulator/adaptogen; GABA_A_ and GABA_P_ receptor agonist; serotonergic action
Amino acids				
L-theanine	50 mg	L-theanine	Anxiety/Insomnia	GABA precursor; Anti-Glutamate; Pro-Dopamine; Pro-Serotonin
Melatonin	1 mg	Melatonin	Insomnia	Action on the circadian rhythm (modulation biological clock)
L-tryptophan	125 mg	L-tryptophan	Anxiety/Insomnia	Precursor melatonin and serotonin
Adenosine	4.25 mg	Adenosine	Insomnia	Increased GABA activity and reduction in cholinergic activity
Glycine	3.10 mg	Glycine	Anxiety/Insomnia	Neuro inhibitory amino acid; NMDA receptor co-agonist
Vitamins				
Vitamin B6	1,68 mg (NVR: 100%)	Vitamin B6	Anxiety/Insomnia	Cofactor for the synthesis of serotonin, norepinephrine, dopamine, melatonin, GABA; HPA axis modulation
Vitamin D	25 mcg (VNR 500%)	Vitamin D	Anxiety/Insomnia	Analgesic, anti-inflammatory, mood modulator effect (vitamin D receptors in the CNS)
Minerals				
Magnesium	225 mg (NVR: 60%)	Magnesium	Anxiety/Insomnia	NMDA receptor antagonist
Zinc	8.7 mg (NVR:87%)	Zinc	Insomnia	NMDA receptor antagonist; cofactor for adenosine synthesis

Statistical analysis

Descriptive statistics were performed. The ANOVA test was performed to evaluate the effect of treatment over time on all clinical and psychometric variables considered, with a significance of p<0.05 (Table 4).

**Table 4:** ANOVA test. dF: degree of freedom. Significance at p<0.05.

Questionnaire	F-value	dF	P value	Post hoc < 0.05
STAY-1	12.058	1.308	<0.001	T0¹T1; T0¹T2
STAY-2	15.118	1.492	<0.001	T0¹T1; T1¹T2; T0¹T2
RSA	9.753	1.911	<0.001	T0¹T1; T0¹T2
PSQI	22.428	1.860	<0.001	T0¹T1; T0¹T2
FSS	1.600	1.670	0.222	
DASS	12.810	1.985	<0.001	T0¹T1; T0¹T2
BDI	10.674	1.727	<0.001	T0¹T1; T0¹T2
SF-36-1	0.406	1.770	0.645	
SF-36-2	2.085	1.870	0.145	
SF-36-3	7.749	1.382	<0.05	T0¹T2
SF-36-4	2.176	1.284	0.152	
SF-36-5	4.613	1.939	<0.05	T0¹T2
SF-36-6	1.956	1.766	0.164	
SF-36-7	0.475	1.735	0.600	
SF-36-8	0.310	1.992	0.735	
ISI	31.533	1.774	<0.001	T0¹T1; T0¹T2
PGI-S	20.954	1.772	<0.001	T0¹T1; T1¹T2; T0¹T2

A linear regression was performed to evaluate the relationship between pre-treatment score (T0) and post-treatment scores (T1 and T2) with significance at p<0.05.

Correlation was performed to evaluate the association between the independent variables, age and level of school education, and test scores with significance at p<0.05. 

## Results

A total of 28 subjects completed the study protocol and filled out the questionnaires in the study periods T0, T1, and T2. 

**Figure 1:**
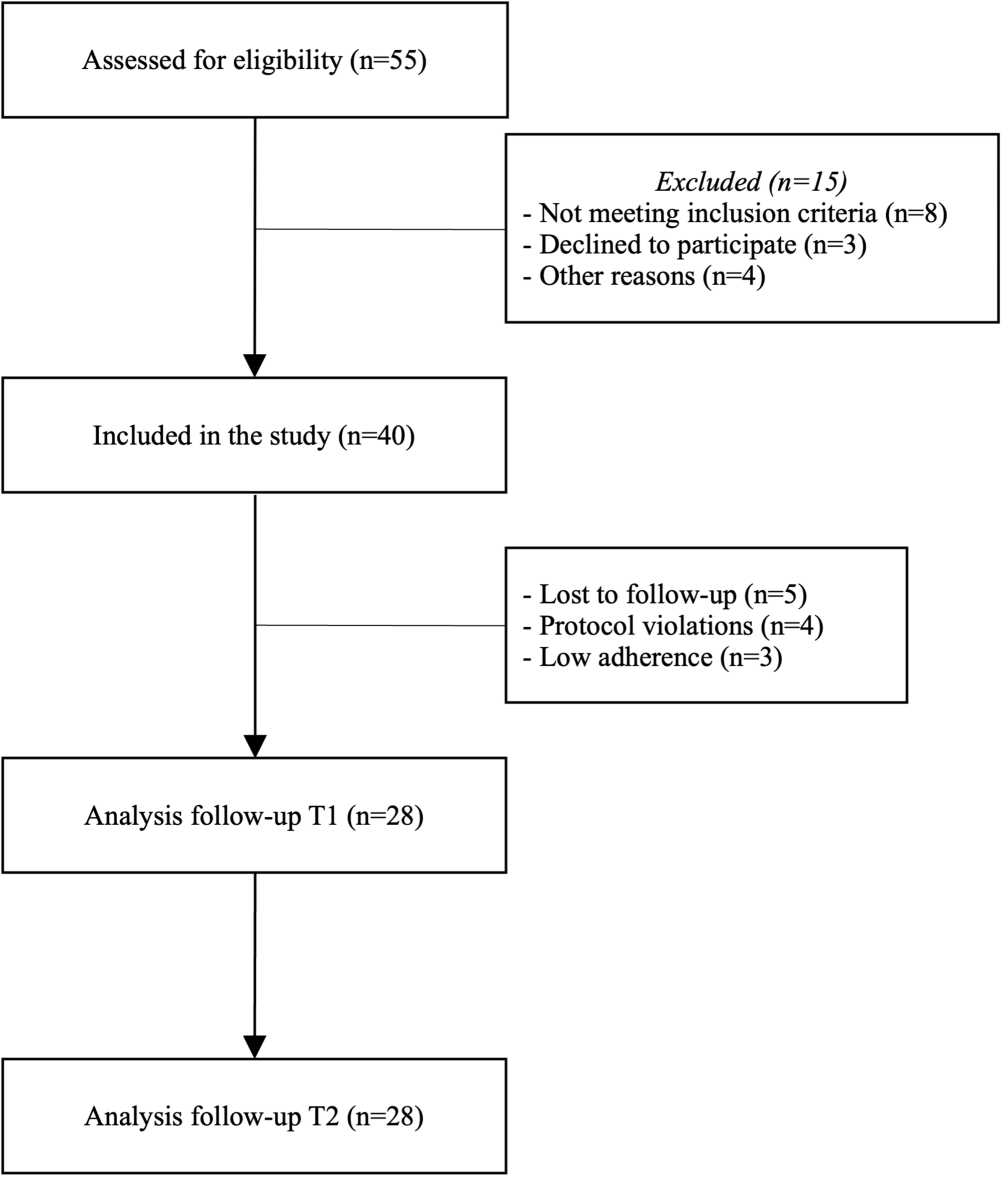
Study patient flow.

The overall sample was composed of 28 subjects (six men, 21.4%; 22 women, 78.6%) with a mean age of 44 ± 7 yrs. Among them, 19% (N = 5) have a middle school diploma, 58% (N = 16) have a high school diploma, and 23% (N = 7) have a university degree. The study patient flow is reported in Figure 1.

The effect of treatment over time was statistically significant for STAY-1, STAY-2, RSA, PSQI, DASS, BDI, ISI, and PGI-S scales and the SF-36 subscales 3 and 5 (p<0.05).

**Figure 2:**
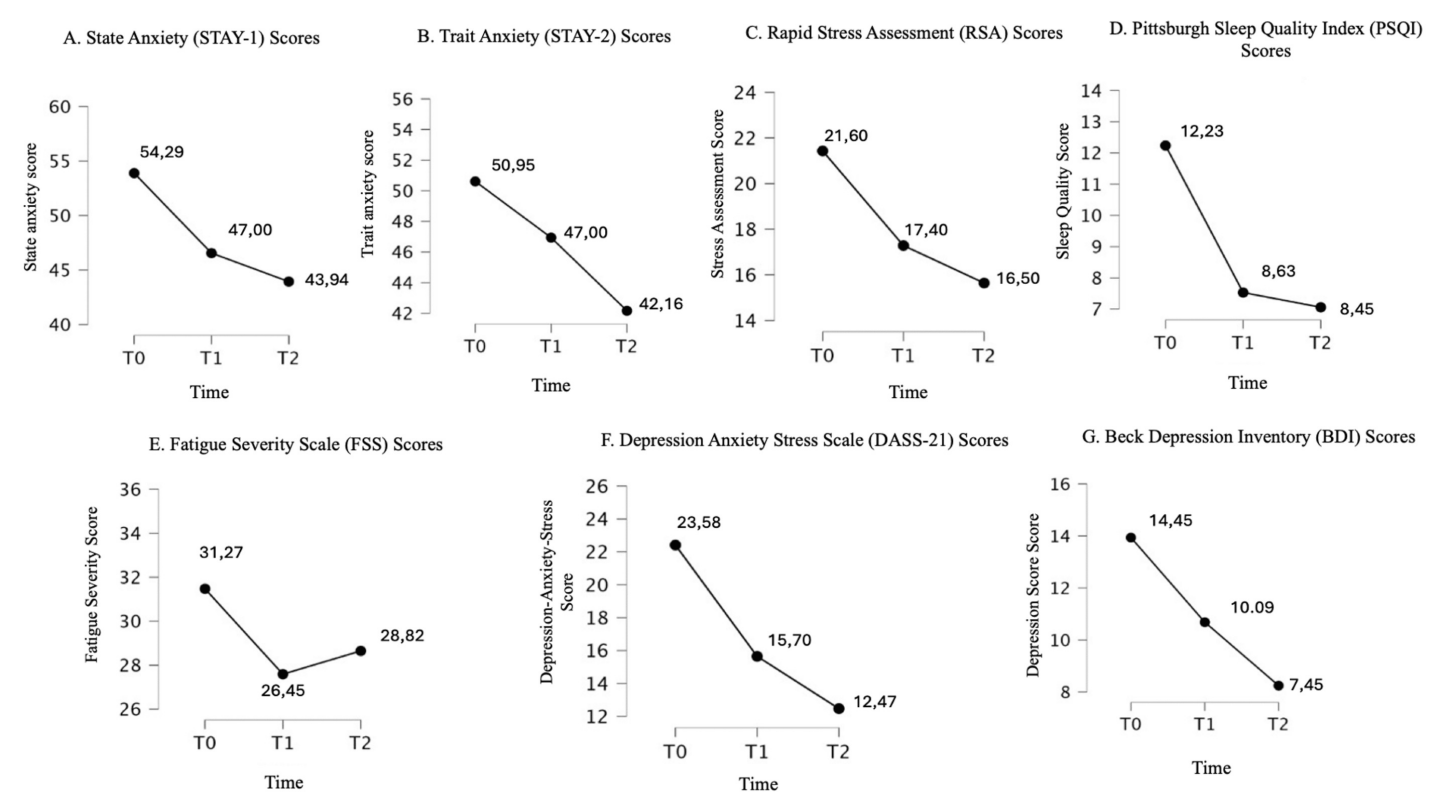
Changes in psychometric and clinical assessment scores from baseline (T0) to eight weeks (T2) A: State Anxiety (STAY-1); B: Trait Anxiety (STAY-2); C: Rapid Stress Assessment (RSA); D: Pittsburgh Sleep Quality Index (PSQI); E: Fatigue Severity Scale (FSS); F: Depression Anxiety Stress Scale (DASS-21); G: Beck Depression Inventory (BDI). The Y-axis represents the respective scores. The X-axis represents time points (T0 = baseline, T1 = four weeks, T2 = eight weeks)

**Figure 3:**
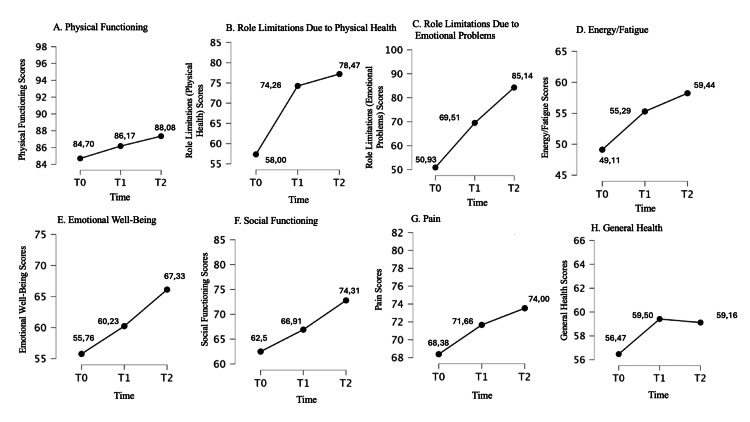
Changes in SF-36 health survey subscale scores from baseline (T0) to eight weeks (T2). A: Physical Functioning; B: Role Limitations Due to Physical Health; C: Role Limitations Due to Emotional Problems; D: Energy/Fatigue; E: Emotional Well-Being; F: Social Functioning; G: Pain; H: General Health. The Y-axis represents the respective subscale scores. The X-axis represents time points (T0 = baseline, T1 = four weeks, T2 = eight weeks).

**Figure 4:**
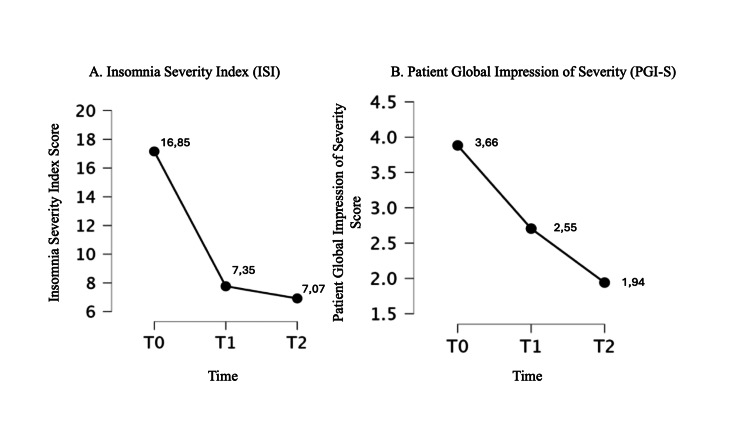
Changes in Insomnia Severity Index (ISI) and Patient Global Impression of Severity (PGI-S) scores from the baseline (T0) to eight weeks (T2). A: Insomnia Severity Index (ISI); B: Patient Global Impression of Severity (PGI-S). The Y-axis represents the respective scores. The X-axis represents time points (T0 = baseline, T1 = four weeks, T2 = eight weeks).

Post hoc test results are reported in Table 4. ANOVA plots for significant outcomes are shown in Figures 2-4.

The linear regression reported a significant association only between T1 and T2 scores (p<0.05). This is probably because the values at T1 and T2 do not deviate significantly from each other, except for the following tests: DASS, PGI-S, and the SF-36 subscales 1 and 2. Therefore, it is not possible to predict from the pre-treatment test (at T0) what the outcome trend might be, except for the SF-36 subscale 8 score. Significant results are reported in Figures 2-4.

No correlations were found with age except for the SF-36 subscale 5 - Pearson (r=0.421; p =0.036) and tau Kendal (0.311; p=0.033) and no correlation was noticed for the level of education.

## Discussion

ID is a widespread disease characterized by subjective difficulty initiating and/or maintaining sleep, with a frequency of manifestation of three times a week and a duration of at least three months. Of note is the impact that this sleep dysfunction has on daily activities from cognitive, psychological, and social perspectives [1].

Although the prevalence of this disorder in the general population is estimated to be between 12 and 20% [17], ID is still underdiagnosed and undertreated. In particular, it has been demonstrated that over 50% of diagnosed patients report having debilitating symptoms even five years after diagnosis [18], supporting the chronic nature of the disease and the need to improve the quality of clinical evaluation and pharmacological and non-pharmacological treatment in affected patients. On the other hand, the chronic nature of ID often requires chronic pharmacological treatment, a therapeutic need that clashes with the limits of the first-line drugs recommended by the EIG [7].

The unmet clinical needs of patients with ID are even more relevant when considering the comorbidity between ID, mental health (depression, anxiety, alcohol dependence), and other chronic diseases such as hypertension, coronary heart disease and metabolic syndrome.

One of the most important topics in ID treatment is the role of a non-pharmacological approach. The importance of this clinical field lies in the fact that it may be able to overcome the time limit established for the first line of drugs recommended for ID such as BZs, BZRAs, and even DORAs.

We have already mentioned the use of CBT-I as a first-line treatment for ID and its limitation due to the lack of availability of psychologists experienced in this clinical field [7].

Taking into account all the above issues, our study aimed to reduce the lack of evidence on the efficacy and safety of dietary supplements for the treatment of ID.

To the best of our knowledge, this is the first study conducted on a sample of patients with a specific diagnosis ID associated with anxiety, on the efficacy and safety of a food supplement composed of multiple "active ingredients" associated with each other to act synergistically on a specific phenotype of patient with ID conducted for eight weeks of treatment. In this dimensional approach to the treatment of patients with ID, our results demonstrated that specifically assembled dietary supplements could exert over time a therapeutic effect not only on sleep quantity and quality (significant reduction of PSQI and ISS scores) but also on other clinical variables that are often comorbid with ID such as anxiety (significant reduction from baseline to weeks 4 and 8 of treatment on STAY-1 and STAY-2, DASS-21 scores), depression (significant reduction from baseline to weeks 4 and 8 of treatment on BDI and DASS-21 scores) and stress (significant reduction from baseline to weeks 4 and 8 of treatment on RSA scores) (Table 4). Interestingly, this overall clinical improvement was perceived by the patient to an increasing extent over the course of treatment, supporting not only the maintenance of clinical benefit but also a clinical response to the compound positively correlated with the duration of treatment (significant reduction from baseline to weeks 4 and 8 and from week 4 to week 8 of treatment on PGI-S scores) (Table 4 and Figure 2). The internal consistency of the beneficial effect of the treatment on the emotional state of the clinical sample was also supported by the improvement of scales 3 and 5 of the SF-36 dedicated to "role limitations due to emotional problems" and "emotional well-being" respectively. As observed for other beneficial effects, also in this case the improvement would be related to the duration of treatment, suggesting once again a positive correlation between treatment time and clinical response (Table 4 and Figure 2). However, during treatment, no effects on the degree of fatigue were observed, although a slight trend was observed after four weeks of treatment (Figure 4). This result could be due to the small size of the clinical sample, which also represents one of the major limitations of the present study. This study employed a single-arm design as an exploratory approach to evaluate the intervention’s efficacy and safety in a real-world setting. While this allowed for preliminary insights, the absence of a control group limits the ability to establish causality. Future research should incorporate randomized controlled trials (RCTs) with placebo or active comparators to strengthen validity and confirm these findings.

According to our primary hypothesis, the significant clinical response observed in the present study could depend on the positive interaction between the clinical phenotype of the study sample and the characteristics of the compound under examination. From this perspective, we preliminarily selected patients with ID associated with a state of anxiety and we chose a compound that acts both on the sleep-wake cycle and on the state of hypervigilance. This overall improvement, which intensified over time, suggests that the compound may be most beneficial for patients with ID and moderate anxiety who experience hypervigilance as part of their symptom profile.

In fact, we hypothesized that a fundamental role in improving both sleep disturbances and anxiety symptoms in our sample study would be played by ashwagandha, an Ayurvedic herb included in the study compound, considered an adaptogenic agent for its property of regulating the stress response and reducing anxiety states (Table 1). The hyperactivation model of ID posits that dysregulation of the sleep-wake cycle is exacerbated by heightened arousal and stress. In this study, components such as ashwagandha, valerian, melatonin, and L-theanine were chosen to target hypervigilance and stress dysregulation through complementary mechanisms.

Indeed, it has been shown that ashwagandha, at a daily dose of 600 mg, could represent a valid therapeutic option in patients with sleep disorders and anxiety. In randomized, double-blind, clinical trials conducted on 60 patients for 10 weeks, ashwagandha was shown to be more effective than placebo on both sleep parameters and anxiety symptoms [19]. Interestingly, as also observed in our pilot study, these beneficial effects were time-related and increased over the course of the study, suggesting that ashwagandha may require time to exert its therapeutic effect on sleep and anxiety disorders [19]. Of course, we are aware that the two studies are not comparable with regard to the type of sleep disorders studied, the study design, and the dosage of ashwagandha used. In particular, in our study, we used a lower dosage of ashwagandha (150 mg once a day) in combination with other food supplements. From this perspective, very few studies have been conducted using the combination of multiple dietary supplements, which suggests the need for research in this field. In an active comparator-controlled study with a polyherbal medicinal product in a sample with a diagnosis of primary insomnia, a combination of three herbal extracts of Valeriana officinalis, Passiflora incarnata, and Humulus lupulus was shown to be as effective as 10 mg zolpidem in the short term (two-week controlled randomized study) [20]. In another four-week pilot study, it was demonstrated that a combination of melatonin, vitamin B6, California poppy extract, passionflower extract, and lemon balm extract could represent a valid therapeutic option in patients with a diagnosis of mild-moderate insomnia [21]. Interestingly, if we put together these last two studies, we can see that the authors used three different food supplements also included in our study compound such as valerian, melatonin, and vitamin B6 (Table 1).

Again, the heterogeneity of the study sample, study design, study duration, and differences in dietary supplements examined make any comparison between studies impossible, but what we want to highlight is the rationale behind using different dietary supplements to treat a complex syndrome like ID. This point represents an important topic of discussion if we consider the last EIG which, for example, does not recommend the use of herbal/phytotherapeutic interventions for the treatment of ID either in the short or long term. This recommendation was made on the basis of a meta-analysis of a few low-quality studies conducted on the efficacy of a single herbal extract and did not discuss the possibility of implementing the knowledge in this field of research by promoting new clinical trials designed to evaluate the efficacy of a combination of different dietary supplements instead of that of a single natural compound [7]. Interestingly, among the plant extracts considered in the EIG meta-analysis, valerian, at doses ranging from 160 to 600 mg per day, was the most important in terms of number of dedicated studies and total number of patients included [7] although its efficacy in this clinical setting has not yet been clearly demonstrated. On the other hand, none of the studies included in these meta-analyses on the efficacy of valerian in the treatment of sleep disorders (17 studies on valerian and five studies on valerian in combination with other plants) were also dedicated to the state of anxiety, often comorbid. From this clinical perspective, although the anxiolytic mechanisms of action of valerian are not fully understood and have been attributed to different constituents of the plant it has been demonstrated that, at least to some extent, these would be modulated by valerenic acid (VA). In particular, VA would exert its action on the GABA-A receptor and therefore on a different site than that of BZs.

Among other nutrients, in addition to ashwagandha and valerian, and despite the lack of good-quality studies, melatonin, L-theanine, magnesium, and glycine have been considered potentially useful for the treatment of sleep disorders and for this reason have been also included in our study compound (Table 1).

Regarding melatonin, it is worth mentioning that the EIG recommends its use in the prolonged-release formulation for short- and long-term therapy (up to three months) in patients over 55 years of age [7]. It is important to specify that this formulation has been approved by the European Medicines Agency for the treatment of insomnia at dosages greater than 1 mg, thus considering it a real drug with a prescription reserved for the medical profession. As a matter of fact, one of the most important problems regarding the use of melatonin in clinical practice is the lack of regulation on quality control on the dosage and purity of the nutrient. To overcome this objective limit, at least on a theoretical level, we used together with melatonin at the dose of 1 mg, a complex of nutrients potentially capable of enhancing its endogenous synthesis such as L-tryptophan, L-theanine, and vitamin B6 (Table 1).

Indeed, as already discussed above, an association between melatonin and vitamin B6 in combination with other plant extracts, has been studied in a sample of patients with mild-moderate insomnia demonstrating therapeutic potential in this specific clinical sample [21]. Interestingly, in a large randomized, placebo-controlled clinical trial of subjects treated with high doses of vitamin B6 (240 mg) for five days, vitamin B6 was shown to increase the amount of dream content recalled by participants, suggesting a more complex mechanism of action of vitamin B6 in regulating sleep architecture [22].

In our study, we observed an improvement in perceived stress in patients with ID, and as discussed, this may be related to ashwagandha's action as a regulator of the hypothalamic-pituitary-adrenal (HPA) axis. It is possible that other dietary constituents of our study compound may also act synergistically in HPA axis regulation, and among these L-theanine is probably one of them. A single dose of 200 mg of L-theanine has been shown to reduce stress in a high-quality randomized controlled clinical trial in healthy subjects using an acute stress model and frontal alpha power recording [23]. The action of L-theanine at a dosage of 200 mg/day has also been demonstrated to be effective in reducing stress-related symptoms measured by the PSQI, the STAI-Y1/Y2, and the self-rating depression scale in a four-week, double-blind, randomized, placebo-controlled study conducted on a sample of healthy subjects [24]. Interestingly, the authors of this latter study used the same psychometric tools to assess the clinical outcome variables that we included in our study, such as PSQI, STAI-Y1/Y2, and a self-administered rating scale for depressive symptoms (Table 3) [24].

Among the constituents of the compound we studied, L-tryptophan could be another actor capable of playing a regulatory role in the physiology of the HPA axis. In fact, in a meta-analysis of 11 RCTs, it was reported that at doses ranging from 0.14 to 3 g per day, L-tryptophan is able to reduce anxiety symptoms and improve positive mood in healthy subjects [25].

There is no doubt that an imbalance between the GABA and glutamate-NMDA receptor systems plays a crucial role in determining the comorbidity of sleep and anxiety disorders [5]. Indeed, the efficacy of BZs and BZRAs as modulatory agents in ID is directly linked to their mechanism of action on GABA receptors. We have already discussed the potential GABAergic mechanism of VA and its rationale for use in the compound we studied. To reinforce this pharmacological mechanism, we also included zinc, magnesium, and glycine in our model natural hypnotic compound.

Zinc is the second most common trace element in the body and participates, as a cofactor, in the regulation of numerous receptors, such as the glutamate α-amino-3-hydroxy-5-methyl-4-isoxazolepropionic acid receptor, and those of dopamine, serotonin, and adenosine, all systems involved in the regulation of the sleep-wake cycle. Deficiency of this trace element together with that of magnesium, has been considered a predisposing factor to depression, as its action on the HPA axis is crucial for regulating cortisol synthesis and inflammation [26]. In this regard, in a randomized clinical trial, the association of zinc and magnesium at doses of 11.5 mg and 225 mg (very similar to the compound we studied) together with 5 mg of melatonin, is effective in improving the quality of sleep and life in patients with primary insomnia [27]. Indeed, it is possible that the reduction in stress, anxiety, and depressive symptoms observed in our clinical sample over the eight weeks of treatment may be due, at least in part, to the antagonism that both zinc and magnesium exert on glutamate/NMDA receptors and consequently on the regulation of HPA axis [26].

Regarding glycine, we recognize that the effective dose of this non-essential amino acid in sleep disturbances and related daily fatigue which makes glycine probably a partial agonist of NMDA receptors, is 3 g/day, significantly higher than that used in this study (Table 1) [28].

In fact, according to the Di Bella model [29] in our study we used low doses of both adenosine and glycine, to improve respectively the absorption of melatonin and the stabilization between melatonin and adenosine, theoretically overcoming in this way the critical issues related to the pharmacokinetics of melatonin together with its low dose allowed in nutraceuticals. On the other hand, to some extent, melatonin has been and still is mistakenly considered a "mere" sleep aid. In reality, melatonin, like vitamin D, is a versatile pleiotropic molecule, capable of acting in a multisystemic manner and synthesized by organs other than the pineal gland, such as the intestine [30]. From this perspective, the improvement of melatonin availability through the synergistic chemical binding with adenosine and glycine could have contributed to improving not only the quality and quantity of sleep disturbances but also to the reduction of perceived stress, anxiety symptoms, and mood regulation observed in our clinical sample, all clinical aspects that melatonin is able to positively modulate [29].

Interestingly, it has been postulated that, just as melatonin is the hormone of darkness, vitamin D is the hormone of light and that the two act synergistically and reciprocally in regulating multiple systemic functions, including inflammation and immune regulation [30]. In fact, like melatonin, vitamin D has a pleiotropic action on multiple physiological systems, including the sleep-wake cycle. From this perspective, although the association of sleep disturbances with low vitamin D levels has been widely demonstrated, it is still not entirely clear what role vitamin D plays in the treatment of sleep disturbances.

Again, we believe it is important to emphasize that the improvement of almost all the clinical variables included in the outcomes of the present study observed in our sample study is likely related more to the summation and even amplification of multiple mechanisms of action exerted by the different dietary supplements included in our natural compound than to the result of a single mechanism of action (Table 1).

We acknowledge that the limitations of this study do not allow us to generalize its findings to all patients with ID. In this regard, the open design of the study, with only one treatment arm, represents the main limitation of our study. On the other hand, in our clinical sample, we observed a progressive improvement over the eight weeks of treatment in almost all clinical variables measured by a validated self-administered questionnaire, suggesting a robust clinical therapeutic effect of the study compound (Figure 2). Certainly, the small sample size does not allow to draw further statistical inferences and, at the same time, the two-month follow-up does not allow to draw conclusions on the long-term efficacy and safety of the compound under study. However, in terms of safety, it is noteworthy that no subjects in the sample study discontinued the trial due to side effects, which at least suggests that this complex dietary supplement does not pose any particular safety concerns (Figure 1). Finally, the monocentric nature of the study could represent a weakness of the experimentation due to the potential non-generability of the results and ability to establish causality, at the same time a strength if we consider the homogeneity of the clinical evaluation and diagnosis performed at the individual level. Future studies should include placebo or active treatment control groups to strengthen the validity of findings and better account for the placebo effect. While this remains a limitation, the study’s eight-week duration makes it less likely that placebo-related benefits would persist throughout, potentially mitigating its influence on the results. In conclusion, the results of the present study represent a proof-of-concept study that supports the clinical utility of a dietary supplement composed of a combination of herbs, trace elements, vitamins, and essential amino acids that act synergistically to improve the sleep-wake cycle, anxiety state and other emotional symptoms in patients with ID.

Multicenter, randomized, double-blind clinical studies using a placebo or an active comparator, conducted on larger clinical samples and over longer periods of time, in association with and without CBT-I and on a more complex clinical sample are needed to understand the real clinical power and safety of this combined food compound.

## Conclusions

In conclusion, the findings of this study provide preliminary evidence supporting the clinical utility of a dietary supplement composed of a synergistic combination of herbs, trace elements, vitamins, and essential amino acids in improving not only the sleep-wake cycle but also anxiety symptoms and overall emotional well-being in patients with ID. The observed significant improvements in sleep quality, anxiety, depression, stress, and quality of life highlight the potential of this natural multicomponent approach as a complementary treatment strategy.

However, given the study’s limitations, these findings should be interpreted with caution. Multicenter, randomized, double-blind clinical studies with placebo or active comparators, conducted on larger and more diverse clinical samples over extended periods, both in combination with and independent of CBT-I, are necessary to determine the full therapeutic potential and long-term safety of this combined nutraceutical formulation.

## Appendices

Abbreviations

BDI: beck depression inventory; BZs: benzodiazepines; BZRAs: benzodiazepine receptor agonists; CBT-I: cognitive behavioral therapy for insomnia; DORAs: dual orexin receptor antagonists; DSM-V: diagnostic and statistical manual of mental disorders; DASS-21: depression and anxiety scale short form; EIG: European Insomnia Guideline; FSS: fatigue severity scale; GABA: γ-aminobutyric acid; HPA: hypothalamic-pituitary-adrenal axis; ID: insomnia disorder; ISS: insomnia severity index; NMDA: N-methyl-D-aspartate; PGI-S: patient global improvement - severity; PSQI: Pittsburgh sleep quality index; RSA: rapid stress assessment; SF-36: short form -36; STAY-1/2: state and trait anxiety inventory; VA: valerenic acid.
